# Targeting Neutrophil Extracellular Traps in Neuroinflammation: A Therapeutic Perspective on Neurodegenerative Diseases

**DOI:** 10.3390/brainsci16080792

**Published:** 2026-07-27

**Authors:** Iolanda Masalskiene Costa, Alexandre Kanashiro, Giovanna Siqueira Favi Barros, Ana Julia Monteiro, Caio Santos Bonilha, Giovane Galdino, Flavio Protasio Veras

**Affiliations:** 1 Multicenter Postgraduate Program in Physiological Sciences, Department of Physiological Sciences, Institute of Biomedical Sciences, Federal University of Alfenas, Alfenas 37133-840, MG, Brazil; iolanda.costa@sou.unifal-mg.edu.br (I.M.C.); giovanna.barros@sou.unifal-mg.edu.br (G.S.F.B.); anajulia.monteiro@sou.unifal-mg.edu.br (A.J.M.); giovane.souza@unifal-mg.edu.br (G.G.); 2 Center for Research in Inflammatory Diseases, Ribeirão Preto Medical School, University of São Paulo, Ribeirão Preto 14040-900, SP, Brazil; 3 Unidade EMBRAPII—FMRP-USP, Ribeirão Preto Medical School, University of São Paulo, Ribeirão Preto 14040-900, SP, Brazil; 4 Institute of Infection, Immunity and Inflammation, University of Glasgow, Glasgow G12 8TA, UK; caio.bonilha@glasgow.ac.uk; 5 Center for Experimental Biology, Laboratory of Neuroimmunobiology of Pain, Federal University of Alfenas, Alfenas 37133-840, MG, Brazil; 6 School of Pharmaceutical Sciences, Federal University of Alfenas, Alfenas 37133-840, MG, Brazil

**Keywords:** neutrophils, neutrophil extracellular traps, neuroinflammation, innate immunity, inflammatory response, neurodegenerative disorders

## Abstract

**Highlights:**

**What are the main findings?**

- NETs contribute to neuroinflammation by promoting blood–brain barrier disruption and the amplification of inflammatory signaling in several neurodegenerative diseases.
- Dysregulated NET formation is increasingly recognized as a mechanistic link between innate immune activation and neuronal injury in neurodegenerative disorders such as Alzheimer’s disease, Parkinson’s disease, and multiple sclerosis.

**What are the implications of the main findings?**

- Targeting the NETosis pathways represents a promising therapeutic strategy to modulate neuroinflammation and reduce neurovascular damage in neurodegenerative disorders.
- Emerging approaches, including PAD4 inhibition, DNase-mediated NET degradation, and modulation of oxidative signaling pathways, may provide new avenues for therapeutic intervention.

**Abstract:**

Neuroinflammation is a complex process involved in the pathogenesis of several neurodegenerative diseases, including Alzheimer’s disease, Parkinson’s disease, multiple sclerosis, Huntington’s disease, and amyotrophic lateral sclerosis. Neutrophils, although traditionally considered peripheral immune cells, have emerged as active participants in the immunopathology of the central nervous system (CNS) through the release of neutrophil extracellular traps (NETs), structures composed of decondensed chromatin embedded with pro-inflammatory proteins. Evidence suggests that NETs play a dual role: they are protective against pathogens but can also induce tissue damage when produced in excess. Several pathways are involved in their formation, including vesicle-mediated release (vital NETs), the lytic NADPH oxidase (NOX)-dependent pathway, and the mitochondrial pathway. Targeting NETs therapeutically, through the use of NETosis inhibitors, NET-degrading strategies, or blockade of neutrophil migration, has shown promise in reducing neuroinflammation/neurodegeneration and improving neurological outcomes in experimental models. This review aims to investigate both the protective and deleterious roles of NETs and how this knowledge may reveal new therapeutic strategies to modulate neurodegenerative diseases and preserve neural integrity, offering valuable insights for potential applications in clinical practice.

## 1. Neuroinflammation, Neutrophils, and NETs in Neurodegenerative Pathology

Neuroinflammation is a complex response to Central Nervous System (CNS) injury that involves increased vascular permeability, activation of glial cells, release of inflammatory mediators, including cytokines and chemokines, leukocyte infiltration, and the generation of reactive oxygen species (ROS) and reactive nitrogen species [1,2,3]. When transient, neuroinflammation largely plays a protective role. However, an increasing number of clinical and preclinical studies have demonstrated that the persistence or dysregulation of this inflammatory process in the CNS is associated with the major pathological factors of various neurological diseases, such as psychiatric conditions, stroke, infectious conditions, and traumatic brain injuries [1]. Particularly, scientific evidence also indicates that neuroinflammation plays an important role in the pathogenesis of neurodegenerative diseases, such as Alzheimer’s, Parkinson’s, and multiple sclerosis, which affect millions of people [2,3].

Although microglia and astrocytes are classically recognized as the main cellular components of neuroinflammation, accumulating evidence indicates that peripheral immune cells, particularly neutrophils, play a key role in shaping the inflammatory landscape of the CNS [4,5]. Neutrophils constitute the first line of defense of the innate immune system, performing an essential role in protecting the host against pathogens. During neuroinflammatory events, the disruption of the blood–brain barrier (BBB) facilitates the recruitment of circulating neutrophils, which, upon activation, release a wide range of cytokines, proteases, and ROS that can either support tissue repair or amplify neural injury [4,5]. Recent studies have identified another crucial defense mechanism, known as neutrophil extracellular traps (NETs). This process depends on the participation of histone-associated proteins, proteases, and the enzymatic activity of myeloperoxidase (MPO) [6,7,8,9,10,11]. However, NETs exhibit dual functionality, exerting protective antibacterial effects but also promoting tissue damage and exacerbating inflammation when formed in excess [6,10,12]. NETs can be released through different pathways: vesicle-mediated release (vital NETs), the nicotinamide adenine dinucleotide phosphate (NADPH) oxidase (NOX)-dependent lytic pathway, and the mitochondrial DNA pathway [6]. All these pathways result from the expulsion of chromatin, associated with cytotoxic proteins, into the extracellular space.

Although several reviews have addressed NETs across heterogeneous CNS insults, including traumatic, neurodegenerative, autoimmune, tumoral, vascular, and infectious [13,14,15,16], a synthesis focusing specifically on chronic neurodegenerative diseases has not yet been provided. This review is organized as an integrated pathway–disease–targeted therapy framework, accompanied by a comparative grading of the strength of evidence across neurodegenerative disorders, highlighting NETs as both biomarkers and potential therapeutic targets in these CNS disorders.

## 2. Molecular Mechanisms of NETosis

NETs are specialized extracellular structures formed by the extrusion of decondensed chromatin from activated neutrophils, composed primarily of nuclear or mitochondrial DNA decorated with histones and a wide array of granular and cytoplasmic proteins, including neutrophil elastase (NE), MPO, cathepsin G, and antimicrobial peptides. These web-like structures create a highly concentrated microenvironment of cytotoxic and proteolytic molecules that enable the physical trapping, immobilization, and neutralization of invading microorganisms, thereby limiting their dissemination and facilitating pathogen clearance. NETs are therefore recognized as a fundamental effector mechanism of innate immunity, particularly effective against pathogens that are too large to be efficiently phagocytosed, such as filamentous fungi and aggregated bacteria [12,17].

NETs have essential physiological functions, such as aiding innate immunity by trapping, immobilizing, and neutralizing a wide variety of pathogens, including fungi, Gram-positive and Gram-negative bacteria, parasites, and viruses [17,18,19,20]. However, not all microorganisms are affected by NETs, as multiple variables influence the activation of this mechanism, including microorganism size. Smaller microbial agents undergo phagocytosis and are weak NET inducers because the resulting phagosome fuses with granules containing essential proteases, rendering them inaccessible for NETosis activation. In contrast, large microorganisms such as the fungus *Candida albicans* stimulate the release of NE into the cytoplasm, initiating the NET mechanism [17,19]. Additionally, NETs play a fundamental role in immunothrombosis, a protective physiological process that is essential not only for preventing blood loss but also for combating viral and bacterial infections by generating a pro-coagulant surface and activating the contact phase of coagulation [21,22,23].

NETs are also responsible for regulating the activation, differentiation, and function of immune cells, including natural killer cells, B, CD4^+^, and CD8^+^ lymphocytes, and even influencing macrophage polarization between M1 and M2 phenotypes [24]. It is important to emphasize that, to maintain tissue homeostasis, there must be a balance between NET formation and clearance. The enzyme Dnase-1 cleaves NET chromatin into smaller fragments, allowing macrophages to phagocytose the remaining parts [25,26,27]. In this process, M2 macrophages, upon interacting with NETs, release chemical mediators that activate M1 macrophages and monocytes, facilitating NET catabolism [26,27,28,29].

Excessive NET formation has been well documented in infectious and systemic inflammatory conditions, including severe SARS-CoV-2 infection, where NETs contribute to endothelial injury, immunothrombosis, and amplification of cytokine-driven inflammation [22,30,31,32,33,34,35,36,37,38,39]. Although COVID-19 is not classified as a neurodegenerative disease, this example illustrates how dysregulated NETosis can shift from a protective antimicrobial response to a pathogenic mechanism of vascular and inflammatory damage. This concept is relevant to neurodegenerative disorders, in which persistent NET formation may similarly contribute to BBB disruption, sustained neuroinflammation, and neuronal injury.

**Figure 1:**
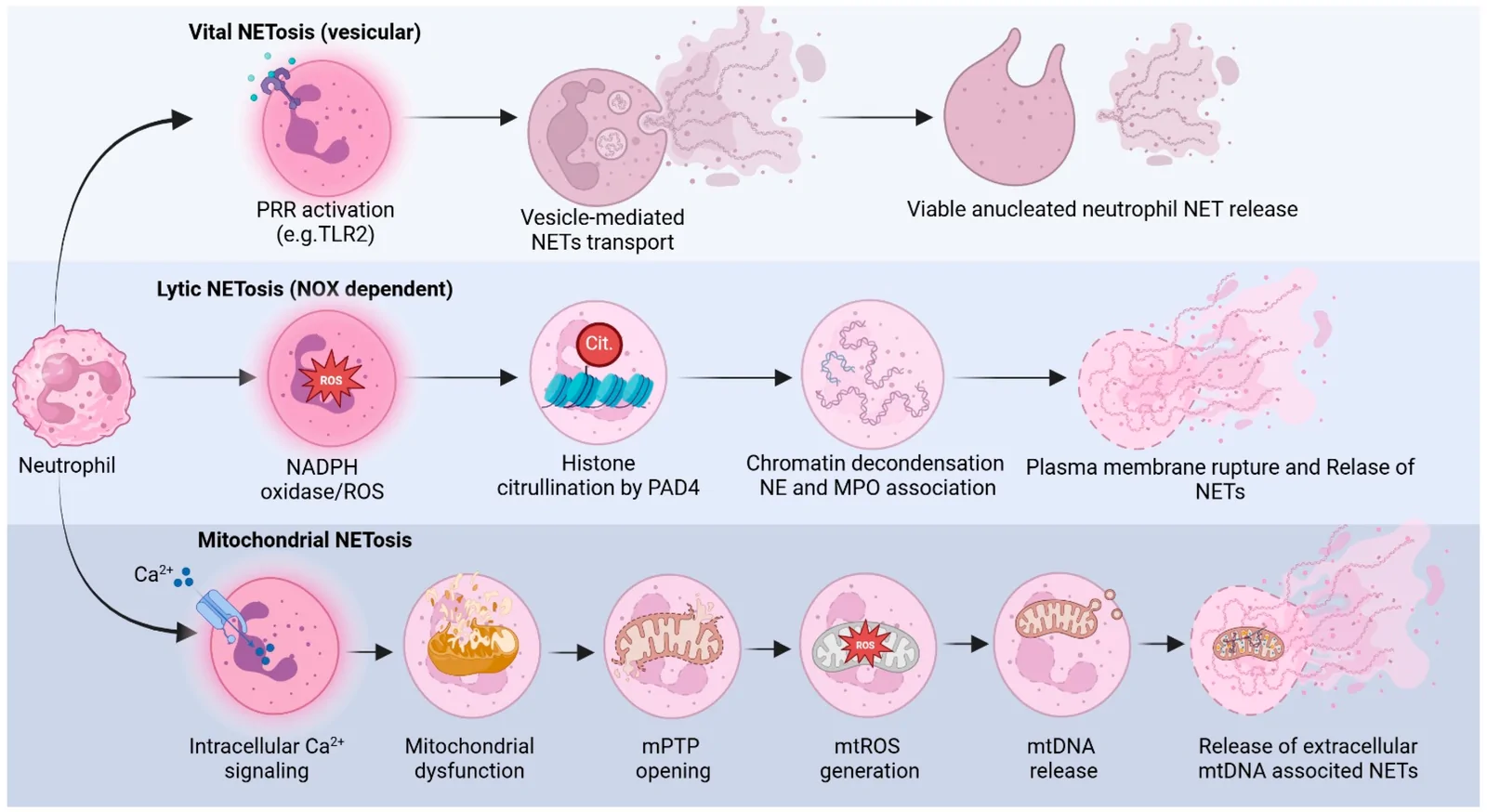
NET-formation pathways. NETs can be generated through three distinct mechanistic pathways that differ in their molecular requirements, kinetics, and cellular outcomes. (**Top**) In the vesicle-mediated pathway (vital NETosis), activation of pattern recognition receptors (PRRs), particularly Toll-like receptors such as TLR2 and TLR4, induces controlled nuclear remodeling and partial chromatin decondensation. Chromatin fragments are packaged into vesicular structures that traffic through the cytoplasm and fuse with the plasma membrane, releasing NET components into the extracellular environment while preserving neutrophil viability and membrane integrity. The resulting cell remains viable but anucleated and retains certain immune functions. (**Middle**) In the lytic or NADPH oxidase (NOX-dependent) pathway, strong inflammatory stimuli activate the NOX complex, leading to the generation of reactive oxygen species (ROS). ROS act as signaling mediators that promote downstream activation of pathways such as MAPKs and the enzyme peptidylarginine deiminase 4 (PAD4), which catalyzes histone citrullination. These events lead to chromatin decondensation, mixing nuclear and granular components, including NE and MPO, and ultimately rupture of the plasma membrane, resulting in cell lysis and extracellular release of NETs. (**Bottom**) In the mitochondrial DNA-dependent pathway (mitochondrial NETosis), NET formation follows a more clearly defined sequence of events. First, neutrophil activation triggers intracellular Ca^2+^ signaling, which promotes mitochondrial dysfunction and the opening of the mitochondrial permeability transition pore (mPTP). The increased mitochondrial permeability promotes mitochondrial ROS (mtROS) generation, which facilitates mtDNA release into the cytosol and extracellular space, where mtDNA associates with histones and granular antimicrobial proteins to form extracellular, mtDNA-associated NETs. This mechanism highlights the metabolic flexibility of neutrophils and represents an alternative pathway of NET formation, independent of nuclear chromatin extrusion. These pathways illustrate the functional heterogeneity of NETosis and underscore the context-dependent mechanisms by which neutrophils deploy extracellular traps during infection and inflammation. Created in BioRender. Veras, F. (2026) https://BioRender.com/6nxc430.

Current evidence supports the existence of three main mechanistic pathways underlying NET formation (Figure 1) which differ in their molecular requirements, kinetics, and cellular outcomes: (i) vesicle-mediated NETs release, in which chromatin-containing vesicles are exported without immediate neutrophil death; (ii) the lytic or NOX-dependent pathway, characterized by extensive chromatin decondensation, plasma membrane rupture, and cell death; and (iii) the mitochondrial DNA pathway, in which mitochondrial DNA is selectively released into the extracellular space in a ROS-dependent manner. Together, these pathways highlight the functional heterogeneity of NETosis and underscore the context-dependent nature of NET formation in inflammatory and infectious settings [14,40,41]. A comparison of the three NET-formation pathways, triggers/stimuli, mechanisms, and predominant biological context, is summarized in Table 1.

### 2.1. Vesicle-Mediated NET Release (Vital NET)

Here, NETs are released through a non-lytic mechanism that preserves neutrophil viability, without loss of plasma membrane integrity or immediate cell death. This process, termed vital NET formation, is considered oxidant-independent, as it does not rely on NOX-derived ROS and occurs independently of mitochondrial DNA release. Instead, this pathway is predominantly mediated by pattern recognition receptors, particularly Toll-like receptor 2 (TLR2), which senses specific microbial components and initiates intracellular signaling cascades leading to controlled chromatin extrusion [42,43].

Upon stimulation by pathogens, the typically segmented, lobulated nucleus of the neutrophil progressively loses its multilobed architecture, becoming rounded, while chromatin undergoes partial decondensation and redistributes uniformly within the nuclear compartment. Subsequently, the localized rupture of the nuclear envelope takes place without compromising overall cellular integrity, allowing chromatin fragments to be packaged into vesicular structures [43]. These DNA-containing vesicles are then trafficked through the cytoplasm and fuse with the plasma membrane, resulting in the extracellular release of chromatin associated with granular antimicrobial proteins. Importantly, following NET release, neutrophils remain viable, retain membrane integrity, and preserve essential effector functions such as chemotaxis and phagocytosis, underscoring the functional significance of this pathway in early host defense responses [41,43,44].

### 2.2. Lytic Pathway (Suicidal/NOX-Dependent Pathway)

In this classical form of NETosis, also referred to as suicidal NETs (NOX-dependent pathway) formation, a wide range of strong agonists, including bacterial components such as lipopolysaccharide (LPS), pharmacological activators like phorbol 12-myristate 13-acetate (PMA), cholesterol crystals, immune complexes, and whole microorganisms (*Candida albicans hyphae*, aggregated bacteria), trigger a robust intracellular oxidative response in neutrophils. This response is mediated by the activation of the NOX complex, which catalyzes the rapid generation of ROS, a central and indispensable event for the initiation and propagation of the NETosis process [45,46,47].

ROS production functions as a pivotal signaling hub that orchestrates multiple downstream events. Elevated intracellular ROS levels promote the activation of mitogen-activated protein kinase (MAPK) signaling pathways, which regulate transcriptional programs and post-translational modifications necessary for NET formation. These signaling cascades lead to profound chromatin remodeling: peptidylarginine deiminase 4 (PAD4) catalyzes histone hypercitrullination, while NE, together with MPO, drives chromatin decondensation, leading to the progressive disassembly of nuclear architecture. Concomitantly, the nuclear envelope and granule membranes disintegrate, allowing granular enzymes and antimicrobial proteins to associate with decondensed chromatin [46,48,49].

As the process advances, loss of plasma membrane integrity ensues, culminating in cell lysis and the release of extensive extracellular networks composed of decondensed nuclear DNA decorated with histones and cytotoxic antimicrobial molecules. These extracellular traps are deposited in the surrounding tissue, where they serve to immobilize and neutralize pathogens but also contribute to local tissue damage and inflammation when formed excessively. Thus, the lytic NOX-dependent pathway represents a highly effective yet potentially detrimental mechanism of innate immune defense, tightly regulated by the intensity and duration of inflammatory stimuli [50].

### 2.3. Mitochondrial DNA Pathway (Mito-NETosis)

In the mitochondrial DNA-dependent pathway of NET formation, neutrophil activation induces a metabolic and bioenergetic reprogramming that culminates in the generation of mitochondrial reactive oxygen species (mtROS). In this context, increased mitochondrial permeability represents a critical upstream event that precedes and sustains mtROS production, distinguishing this pathway from the classical NADPH oxidase-dependent mechanism [51]. Recognition of specific pathogens or inflammatory stimuli initiates this process through a rapid elevation of intracellular calcium levels, which serves as a key second messenger linking surface receptor activation to mitochondrial dysfunction [51].

The rise in cytosolic calcium concentration promotes an abrupt increase in the permeability of the inner mitochondrial membrane (IMM), driven by the reversible opening of a non-specific mitochondrial permeability transition pore (mPTP). This transient pore formation disrupts mitochondrial homeostasis, facilitating ion fluxes and mitochondrial depolarization while preserving short-term cellular viability [51,52]. Importantly, mPTP opening constitutes a highly efficient trigger for mtROS generation, as altered electron transport chain activity under these conditions favors excessive electron leakage and ROS production.

The accumulation of mtROS, in turn, amplifies oxidative stress within the neutrophil and further promotes mPTP opening, establishing a self-sustaining positive feedback loop. This vicious cycle intensifies mitochondrial dysfunction and supports the release of mitochondrial DNA into the cytosol and extracellular space, where it associates with histones and granular proteins to form NET structures [51,53]. Through this mechanism, mito-NETosis enables neutrophils to deploy extracellular traps in a manner mechanistically distinct from the release of nuclear chromatin-based NETs, highlighting the metabolic flexibility and functional diversity of NET-formation pathways.

## 3. Implications of NETs in Neurodegenerative Diseases

The integrity of the BBB, formed by brain microvascular endothelial cells (BMECs) connected by adherens and tight junctions, is essential for CNS homeostasis. Its loss of integrity, together with sustained neuroinflammation, represents a central event in the pathogenesis of neurodegenerative diseases. Under physiological conditions, neutrophils are rarely detected in the brain parenchyma because they cannot readily cross this highly selective barrier [5]. However, during neuroinflammatory processes, BBB permeability may increase, primarily due to the release of pro-inflammatory cytokines by activated astrocytes and microglia. These mediators promote the upregulation of adhesion molecules, such as intercellular adhesion molecule-1 (ICAM-1), on BMECs, thereby facilitating neutrophil adhesion and extravasation into the CNS [54].

The interaction between neutrophils and BMECs promotes neutrophil activation that, mediated by β2 integrins, including Lymphocyte-Function-Associated Antigen (LFA)-1 and mac-1, enhances oxidative stress, induces NET formation, and triggers the release of NE, both freely and embedded within NET structures. NE-mediated proteolysis degrades junctional proteins such as VE-cadherin and β-catenin, further compromising BBB integrity [55,56]. Pharmacological inhibition of NE using agaphelin has been shown to significantly reduce BBB permeability, infarct volume, and mortality, while improving neurological outcomes in murine models of ischemic stroke [57].

In addition to NE, matrix metalloproteinase-9 (MMP-9), associated with NETs, degrades type IV collagen in the basal lamina of cerebral microvessels, exacerbating BBB disruption [58]. Histones present in NET structures further increase vascular permeability, particularly in hippocampal regions, by destabilizing adherens and tight junctions [59]. In parallel, extracellular DNA derived from NETs acts as a potent inflammatory stimulus by activating the cGAS–STING signaling pathway, promoting the production of type I interferons and pro-inflammatory cytokines by M1-polarized microglia, which intrinsically express cGAS [60,61]. Notably, experimental depletion of neutrophils or inhibition of PAD4, an enzyme essential for NETosis, significantly attenuates type I interferon responses and improves neurological outcomes in stroke [62].

### 3.1. Alzheimer’s Disease

Alzheimer’s disease is a progressive neurodegenerative disorder characterized by cognitive decline, accumulation of amyloid-β (Aβ) peptides, formation of neurofibrillary tangles, neuronal loss, and BBB dysfunction, which impairs Aβ clearance and favors its cerebral accumulation [63]. A pronounced neuroinflammatory state marks the disease course, and accumulating evidence indicates that interactions between neutrophils and microglia, in addition to NET release, contribute to Alzheimer’s disease pathogenesis. Experimental studies have shown that neutrophil accumulation in the brain precedes cognitive impairment in murine models of Alzheimer’s disease, suggesting that innate immune activation is an early event in disease progression [64,65]. More recently, transcriptomic and histopathological analyses have confirmed the presence of NET-associated molecular signatures in Alzheimer’s disease brains, particularly in regions with high amyloid burden and vascular dysfunction, reinforcing the contribution of NETosis to chronic neuroinflammation and neurovascular injury [5,66].

Aβ peptides promote neutrophil adhesion and transmigration by activating LFA-1 and Mac-1, thereby stimulating the release of inflammatory mediators and NETosis. Interestingly, NET-associated neutrophil elastase may further digest and fragment Aβ fibrils, generating short neurotoxic species and amplifying neuroinflammation [64,67]. The presence of NETs in brain tissue correlates with Aβ burden and neurofibrillary tangle density, thereby increasing ROS production and neuronal damage [67,68]. In addition, NET-derived components such as MPO and histones directly compromise BBB integrity and cerebral microcirculation, contributing to hypoperfusion and cognitive decline [66]. Clinically, elevated systemic inflammatory markers such as ICAM-1 and C-reactive protein (CRP) [69,70], in addition to increased levels of neutrophil-associated inflammatory proteins such as MPO and MIP-1β [71], are correlated with dementia severity and disease progression. Persistent neutrophil hyperactivation and NET formation may promote premature apoptosis, sustain microglial activation, and perpetuate inflammatory cascades, thereby accelerating Alzheimer’s disease pathology [13]. Therefore, mechanistically, the NET formation reported in Alzheimer’s disease is most consistent with the NOX/PAD4-dependent suicidal pathway (Section 2.2): amyloid-β and the complement anaphylatoxin C5a recruit and activate neutrophils, and PAD4-dependent, citrullinated histone NET signatures are detectable in amyloid-laden, vascularly compromised regions [15,16].

### 3.2. Parkinson’s Disease

Parkinson’s disease is a chronic neurodegenerative disorder characterized by progressive loss of nigrostriatal dopaminergic neurons and pathological accumulation of α-synuclein [72]. Although the relationship between NETs and Parkinson’s disease has historically been less explored than in Alzheimer’s disease, growing evidence supports the involvement of innate immune dysregulation and neutrophil activation in Parkinson’s disease pathophysiology [73]. Clinical studies have consistently demonstrated that patients with Parkinson’s disease exhibit a significantly higher neutrophil-to-lymphocyte ratio (NLR) than healthy individuals, supporting the presence of a systemic inflammatory state associated with the disease [74]. Recent transcriptomic and immune profiling analyses further identified enrichment of NET-associated genes and neutrophil activation pathways in Parkinson’s disease, correlating with immune infiltration patterns and clinical subtypes of the disease [75,76].

Moreover, Parkinson’s disease patients exhibit increased neuronal nitric oxide synthase (nNOS) expression in circulating neutrophils, leading to elevated nitric oxide production [77]. Nitric oxide reacts with superoxide anions to generate peroxynitrite, a potent oxidant known to exacerbate inflammation and trigger the release of dissociated NETs, thereby amplifying oxidative and inflammatory stress [78]. These mechanisms suggest that NETs may contribute to dopaminergic neurodegeneration by sustaining chronic innate immune activation, oxidative damage, and neurovascular dysfunction, supporting the hypothesis that NETosis participates in Parkinson’s disease progression rather than representing a secondary epiphenomenon.

In Parkinson’s disease, current evidence points to an oxidant-driven process rather than a defined canonical route: nNOS-derived nitric oxide and peroxynitrite in circulating neutrophils promote the release of dissociated NETs, consistent with ROS-dependent (NOX/mtROS) formation (Section 2.2 and Section 2.3). However, the predominant pathway has not been experimentally resolved [14,78].

### 3.3. Multiple Sclerosis

Multiple sclerosis is a chronic autoimmune disease of the CNS, characterized by demyelination and neuronal damage [79]. Neutrophil infiltration into the CNS plays a central role in multiple sclerosis pathogenesis, contributing to BBB disruption through the secretion of inflammatory mediators, such as TNF-α, IL-6, IL-12, IL-1β, interferon (IFN)-γ, and IL-17, the latter particularly facilitating the migration of inflammatory cells and modulating the production of chemokines such as CXCL1 and CXCL2 [80,81,82]. Elevated levels of circulating NETs have been detected in the peripheral blood of multiple sclerosis patients, particularly in males [83]. Neutrophils may also exert direct neurotoxic effects via CXCR2 receptor signaling, which promotes ROS generation, and genetic or pharmacological inhibition of this pathway has been shown to prevent neuronal death [84,85].

In relapsing–remitting multiple sclerosis, neutrophils display increased resistance to apoptosis, possibly driven by inflammatory cytokines such as IL-1β, IL-6, and IFN-γ, along with elevated expression of surface markers including TLR2, CD43, FPR1, and CXCR1 [86,87]. These primed neutrophils exhibit enhanced degranulation, oxidative burst, and NET release. Importantly, NET formation has been directly associated with BBB dysfunction and endothelial injury in multiple sclerosis, linking innate immune activation to neurovascular damage [88,89]. Nevertheless, the precise spatiotemporal mechanisms by which NETs contribute to lesion formation and disease progression remain to be fully elucidated. Together, these studies suggested that, in multiple sclerosis, primed, apoptosis-resistant neutrophils with an enhanced oxidative burst engage both vital and NOX-dependent suicidal NETosis (Section 2.1 and Section 2.2); CXCR2-mediated ROS generation links neutrophil activation to neurotoxicity and BBB injury [15,90].

### 3.4. Huntington’s Disease

Huntington’s disease is a genetic neurodegenerative disorder characterized by progressive motor dysfunction, cognitive decline, and psychiatric manifestations [91]. Although the role of NETs in Huntington’s disease remains less defined than in other neurodegenerative diseases, systemic inflammation and neutrophil-related mediators appear to contribute to disease pathology. Elevated plasma levels of IL-6, MMP-9, VEGF, and TGF-β1 have been reported in patients with Huntington’s disease, suggesting an inflammatory milieu that may promote BBB disruption, thereby facilitating neutrophil infiltration into the CNS and subsequent neutrophil activation [92]. Collectively, these alterations may create a permissive environment for NET-associated tissue damage, but no specific NETosis pathway has yet been characterized [88].

Interestingly, IL-6 deficiency in murine models of Huntington’s disease exacerbated disease symptoms, indicating that tightly regulated inflammatory signaling is required for physiological balance and neuronal survival [93]. This observation highlights the complexity of neutrophil-driven immune responses in Huntington’s disease. It suggests that dysregulated NET formation, rather than inflammation per se, may represent a pathogenic mechanism contributing to disease progression.

### 3.5. Amyotrophic Lateral Sclerosis

Amyotrophic lateral sclerosis is a neurodegenerative disease characterized by progressive motor neuron degeneration and dysregulation of inflammatory pathways, accompanied by increased levels of pro-inflammatory cytokines and, more recently, altered neutrophil functions, particularly NET formation [94,95]. Formation of the IL-6/sIL-6R complex, resulting from sIL-6R release by neutrophils and monocytes, activates pro-inflammatory intracellular pathways, promoting immune cell extravasation and BBB disruption [96,97]. Amyotrophic lateral sclerosis progression has been associated with an increased neutrophil-to-lymphocyte ratio and the infiltration of neutrophils and mast cells into skeletal muscle, contributing to denervation and muscle atrophy through NET formation and ROS release [98,99,100]. CD16 expression in neutrophils also correlates with disease severity and progression, and evidence suggests that sex-related factors modulate the immune response, with differences in the association between peripheral neutrophil levels and survival in men and women with amyotrophic lateral sclerosis [101,102,103]. In amyotrophic lateral sclerosis, NET formation appears to be primarily driven by ROS and has been documented predominantly in peripheral tissues, including skeletal muscle and the neuromuscular junction. However, whether a specific intracellular signaling pathway predominates within the CNS remains to be determined [14,88].

Collectively, the evidence discussed above indicates that, although the degree of NET involvement varies across neurodegenerative diseases, a convergent pathogenic axis emerges that links BBB dysfunction, sustained neuroinflammation, and progressive neuronal injury. In Alzheimer’s disease and multiple sclerosis, mechanistic and experimental data provide stronger support for a direct contribution of NETosis to disease progression. In contrast, in Parkinson’s and Huntington’s disease, current findings suggest an emerging but still incompletely characterized role of neutrophil activation and NET formation. In amyotrophic lateral sclerosis, peripheral neutrophil activation and inflammatory signaling appear to correlate with disease severity and progression. A comparative overview of the level of evidence, principal mechanisms, and CNS consequences associated with NET involvement in these disorders is summarized in Table 2.

## 4. NETs as a Therapeutic Target

The formation of NETs has been widely associated with the amplification of neuroinflammation and the worsening of neurological deficits in CNS diseases, as it contributes to BBB disruption, the release of pro-inflammatory cytokines, oxidative stress, thrombosis, neuronal death, and cognitive dysfunction [5,14]. Thus, targeting neutrophils or inhibiting NETosis emerges as a promising therapeutic strategy.

**Figure 2:**
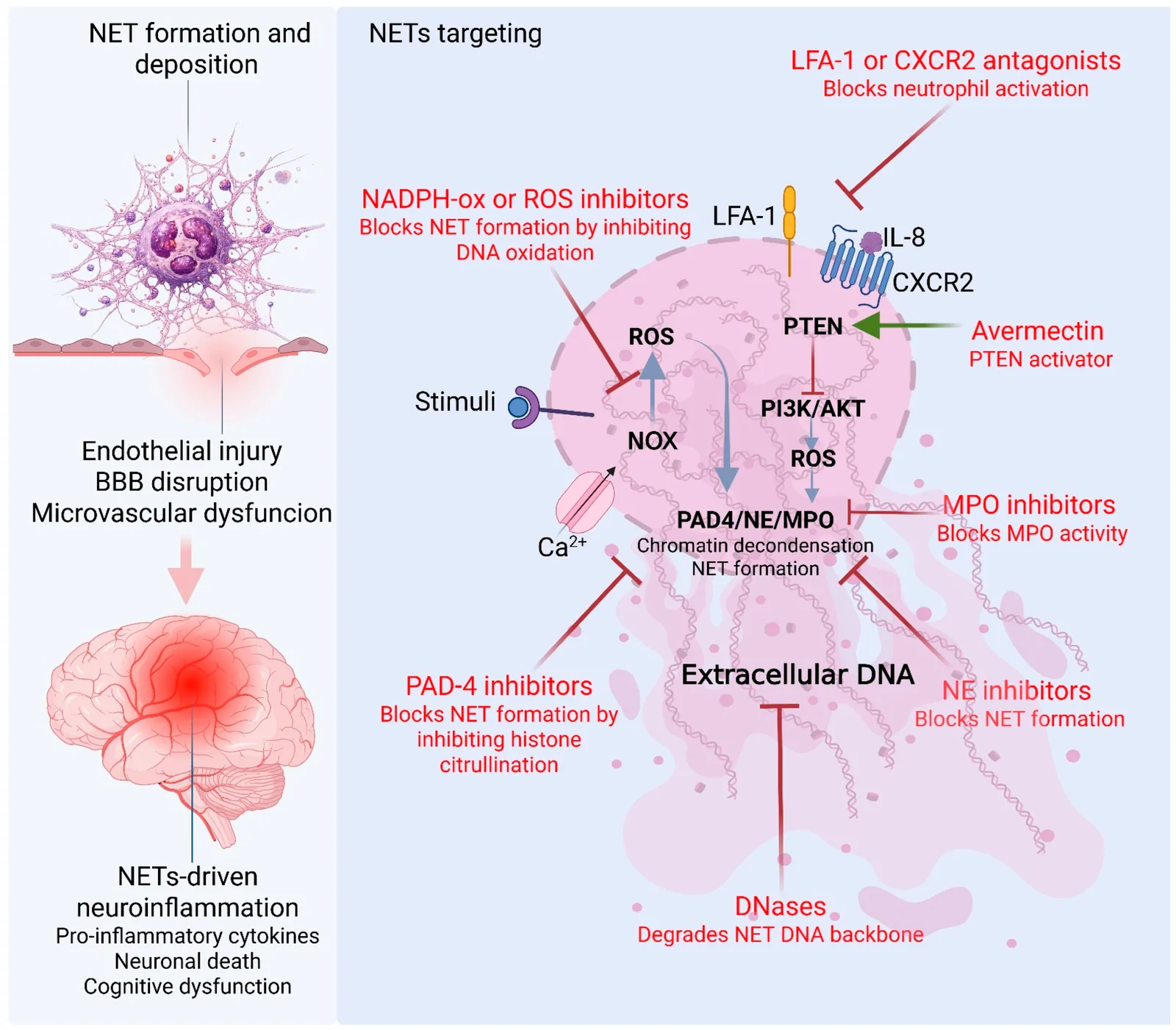
Major pathways of NET formation and therapeutic targeting strategies in CNS disorders. This schematic illustrates the major molecular pathways involved in neutrophil extracellular trap (NET) formation and their contribution to central nervous system (CNS) injury. Upon inflammatory stimulation, activation of NADPH oxidase (NOX) promotes the generation of reactive oxygen species (ROS), which act as key upstream mediators of NETosis. Elevated ROS levels trigger activation of peptidylarginine deiminase 4 (PAD4), neutrophil elastase (NE), and myeloperoxidase (MPO). These enzymes cooperate to induce chromatin decondensation through histone citrullination and proteolytic remodeling, culminating in the release of extracellular DNA–protein complexes known as NETs. Extracellular NET deposition contributes to endothelial injury, BBB disruption, and microvascular dysfunction, thereby amplifying neuroinflammation within the CNS. NET-driven pathology is associated with increased release of pro-inflammatory cytokines, neuronal death, and cognitive dysfunction. Therapeutic strategies targeting NETosis include inhibition of NOX or ROS production to attenuate upstream oxidative signaling; PAD4 inhibitors to block histone citrullination and chromatin decondensation; NE inhibitors to interfere with chromatin remodeling; MPO inhibitors to reduce oxidative amplification of NET formation; and DNases (deoxyribonucleases), which enzymatically degrade the extracellular DNA backbone of established NETs. Collectively, these approaches aim to disrupt key biochemical circuits sustaining NET-mediated vascular and neuroinflammatory damage in neurodegenerative disorders. Created in BioRender. Veras, F. (2026) https://BioRender.com/ed0o57m.

NET formation involves signaling cascades dependent on NOX, ROS, NE, and PAD4, which drive chromatin decondensation and the release of cytotoxic DNA–protein structures into the inflammatory microenvironment. Recent studies demonstrate that pharmacological or genetic modulation of key regulators of NETosis, in addition to inflammatory receptors and signaling mediators that promote neutrophil infiltration into the CNS (e.g., TLR4, IL-1β, and LFA-1), reduces NET formation and attenuates neuroinflammation and the subsequent injurious cascade, leading to improved functional outcomes in experimental stroke and chronic neuroinflammation [14,40]. These findings underscore that effective therapeutic strategies must extend beyond a mere inhibition of NET formation and instead aim to disrupt the biochemical and cellular circuits that sustain pro-inflammatory and pro-thrombotic signaling within neural tissue (Figure 2).

Studies have shown that neutrophil depletion or blockade of their migration, such as through LFA-1 inhibition, reduces NET formation and attenuates neuropathology in murine models of Alzheimer’s disease, improving memory even in advanced stages of the disease [16]. However, given the importance of neutrophils in immunity, complete depletion may be counterproductive. Therefore, more specific targets have been investigated, such as Dnase-1, which directly degrades the DNA backbone of NETs, and PAD4 inhibitors, which block the enzyme responsible for chromatin decondensation and NET release, thereby reducing neuroinflammation, neuronal injury, and microvascular dysfunction [106,107,108].

Recently, it was demonstrated that activated platelets after ischemic stroke induce NETosis via HMGB1, exacerbating damage [109]. Interestingly, the same group identified a natural inhibitor of NET formation present in umbilical cord blood, termed nNIF, which represents a potential therapeutic agent. These findings reinforce the critical role of NETs in the pathophysiology of neurological disorders and highlight targeted approaches to their modulation as innovative therapeutic alternatives.

Furthermore, the formation of NETs largely depends on the generation of ROS, which are produced in large quantities during cerebral ischemia/reperfusion [110,111]. The relationship between oxidative stress and NET formation is mediated by NE and MPO, which intensifies chromatin decondensation [110,112]. In this context, potential therapeutic targets include modulating MPO via macrophage migration inhibitory factor (MIF), which directs MPO activity toward the production of antimicrobial hypochlorous acid, thereby inhibiting NET formation and oxidative stress [110,113]. Another approach involves the use of avermectin, which has been shown to suppress NET release and MPO expression by promoting demethylation of PTEN, a tumor suppressor gene [110,114]. Likewise, NET inhibition is also modulated through intracellular pathways related to neutrophil activation, such as the PI3K/Akt pathway [110,115].

Qingchun M. et al. (2024) presented the formulation of a nanoparticle, T-GSK, consisting of a peptide-functionalized polymer designed to encapsulate the drug GSK484 (GSK), which inhibits PAD4, an essential component of NETosis [116]. This strategy was proposed as a therapeutic approach to reduce NET formation in traumatic brain injury, as NETs exacerbate acute neurological damage by increasing local ROS levels [116,117]. The results of this study indicate that elevated ROS production following traumatic brain injury promotes cleavage of oxidative-stress-sensitive bonds within T-GSK, enabling controlled release of GSK at the lesion site and thereby favoring neuroprotection.

The principal therapeutic strategies can be organized according to the pathway step targeted (Figure 2): upstream oxidative signaling shared by the NOX-dependent and mitochondrial pathways (NOX/ROS and MPO modulators); the citrullination and chromatin remodeling steps specific to the suicidal NOX-dependent pathway (PAD4 and NE inhibitors); the already-released NET scaffold common to all pathways (DNase-1); and the pre-NETotic recruitment and activation stage (LFA-1 and CXCR2 blockade) [15,40,88].

Therefore, targeting neutrophils or inhibiting the detrimental effects of NETosis represents a promising therapeutic strategy for treating CNS disorders. However, several challenges must be overcome before NET-targeted therapies can be translated into clinical practice. A major limitation is the considerable complexity and heterogeneity of NET formation and function across different pathological conditions, making the development of broadly effective therapeutic approaches particularly challenging. Moreover, although studies using animal models and in vitro systems have provided important mechanistic insights, these preclinical models do not fully recapitulate the complexity of NET-associated pathology in humans. Consequently, further translational studies and well-designed clinical investigations are required to validate the therapeutic potential of NET-targeted interventions in CNS disorders [118].

## 5. Conclusions

The evidence summarized in this review reinforces the view that NETs are a key link between innate immunity and neurodegenerative processes. Although they play an essential role in host defense against microorganisms, their excessive formation is associated with BBB disruption, the amplification of the neuroinflammatory response, and the progression of neuronal injury. These events contribute to the pathogenesis of several neurodegenerative diseases, including Alzheimer’s, Parkinson’s, multiple sclerosis, Huntington’s disease, and amyotrophic lateral sclerosis. A detailed understanding of the mechanisms regulating NET formation offers new therapeutic possibilities—PAD4 inhibition, extracellular DNA degradation, and blockade of neutrophil migration—which have demonstrated efficacy in mitigating inflammation and preserving neural integrity in experimental models. Thus, controlling NETosis and modulating neutrophil activity emerge as potential therapeutic targets to reduce the deleterious effects of neuroinflammation and protect neural integrity, paving the way for innovative approaches in the treatment of neurodegenerative diseases.

## Figures and Tables

**Table 1:** Comparison of the three NET-formation pathways: triggers, mechanisms, and predominant biological context.

Feature	Vital (Vesicular)	Suicidal/Lytic (NOX-Dependent)	Mitochondrial (Mito-NETosis)
Prototypical triggers	TLR2/TLR4 PAMPs; *S. aureus*; low-dose LPS; complement	PMA; sustained LPS; cholesterol crystals; immune complexes; large pathogens (*C. albicans* hyphae, aggregated bacteria)	GM-CSF priming + C5a or LPS; Ca^2+^ ionophores; sterile/ischemic stimuli
Kinetics	Minutes (rapid)	Hours (slow)	Rapid
ROS source	Oxidant-independent (NOX-independent)	NOX-derived ROS	Mitochondrial ROS (NOX-independent)
Core signaling	PRR signaling → vesicular chromatin export	NOX → ROS → MAPK → PAD4 citrullination → NE/MPO remodeling	Ca^2+^ → mitochondrial dysfunction → mPTP opening → mtROS generation → mtDNA release
DNA source	Nuclear (vesicle-packaged)	Nuclear (lytic)	Mitochondrial
Cell fate	Viable, membrane intact	Lytic cell death	Viable (short-term)
Predominant context	Early, rapid antibacterial defense	Strong/persistent or sterile (crystals, immune complexes) inflammation; chronic neuroinflammation	Sterile inflammation, ischemia–reperfusion, metabolic/oxidative stress

**Table 2:** Comparison of neurodegenerative diseases in terms of NET involvement.

Disease	NETs Involvement	Mechanisms and Evidence	Consequences for the CNS	References
**Alzheimer’s disease**	Robust evidence of NET involvement	Aβ peptide induces neutrophil adhesion and transmigration via LFA-1 and Mac-1, NETosis, and cytokine release (TNF-α, IL-6, IL-8, IL-1β). NETs correlate with Aβ load and neurofibrillary tangles and generate ROS. Elevated levels of ICAM-1, MPO, and CRP, along with activated neutrophils, are observed.	Aβ fragmentation, exacerbated neuroinflammation, neuronal damage, and cognitive decline.	[5,13,14,40,63,65,66,68,71,90,104]
**Parkinson’s disease**	Suggested involvement is still poorly established	Parkinson’s disease patients present with an elevated neutrophil-to-lymphocyte ratio and higher neutrophil NOS expression, leading to peroxynitrite production that induces the release of dissociated NETs.	Aggravation of inflammation and possible acceleration of dopaminergic degeneration.	[14,40,74,75,76,78,105]
**Multiple sclerosis**	Strong association with pathogenesis	Neutrophil infiltration and cytokine release (IL-1β, IL-6, TNF-α, IFN-γ, IL-17), increasing chemokine production. Resistance to apoptosis and increased NETosis, along with upregulated expression of CXCR1, FPR1, and TLR2.	BBB dysfunction, neuronal death, and demyelination.	[14,40,79,80,81,82,83,84,85,86,87,88,89]
**Huntington’s disease**	The relationship is still incipient and indirect	Increased plasma IL-6, MMP-9, VEGF, and TGF-β1 suggest neutrophil activation. The absence of IL-6 worsens disease in murine models, suggesting a regulatory role for IL-6.	Possible inflammatory contribution, NET role is uncertain.	[14,40,91,92,93]
**Amyotrophic lateral sclerosis**	Significant relationship with disease progression	Formation of the IL-6/sIL-6R complex, derived from neutrophils and monocytes, activates inflammation. NETs contribute to inflammation and muscle damage. Neutrophil infiltration, increased CD16 expression, and a higher neutrophil-to-lymphocyte ratio.	BBB impairment, denervation, muscle atrophy, and progression of motor degeneration.	[14,40,96,97,98,99,100,101,102,103]

## Data Availability

No new data were created or analyzed in this study. Data sharing is not applicable to this article.

## Abbreviations

Blood–brain barrier, BBB; brain microvascular endothelial cells, BMECs; C-reactive protein, CRP; central nervous system, CNS; granulocyte–macrophage colony-stimulating factor, GM-CSF; intercellular adhesion molecule-1, ICAM-1; interleukin, IL; lipopolysaccharide, LPS; lymphocyte-function-associated antigen-1, LFA-1; interferon, IFN; matrix metalloproteinase-9, MMP-9; mitochondrial DNA, mtDNA; mitochondrial permeability transition pore, mPTP; mitochondrial ROS, mtROS; mitogen-activated protein kinase, MAPK; myeloperoxidase, MPO; nicotinamide adenine dinucleotide phosphate, NADPH; NADPH oxidase, NOX; neuronal nitric oxide synthase, nNOS; neutrophil elastase, NE; neutrophil extracellular traps, NETs; pattern-recognition receptor, PRR; pathogen-associated molecular patterns, PAMPs; peptidyl arginine deiminase 4, PAD4; phorbol 12-myristate 13-acetate, PMA; reactive oxygen species, ROS; Toll-like receptors, TLR.
